# Supplementary material for: Evaluating possible maternal effect lethality and genetic background effects in Naa10 knockout mice
Source: PLoS One. 2024 May 7;19(5):e0301328. doi: 10.1371/journal.pone.0301328 (PMC11075865; doi:10.1371/journal.pone.0301328)
Supplement: S1 Raw images — (PDF) [file pone.0301328.s002.pdf]

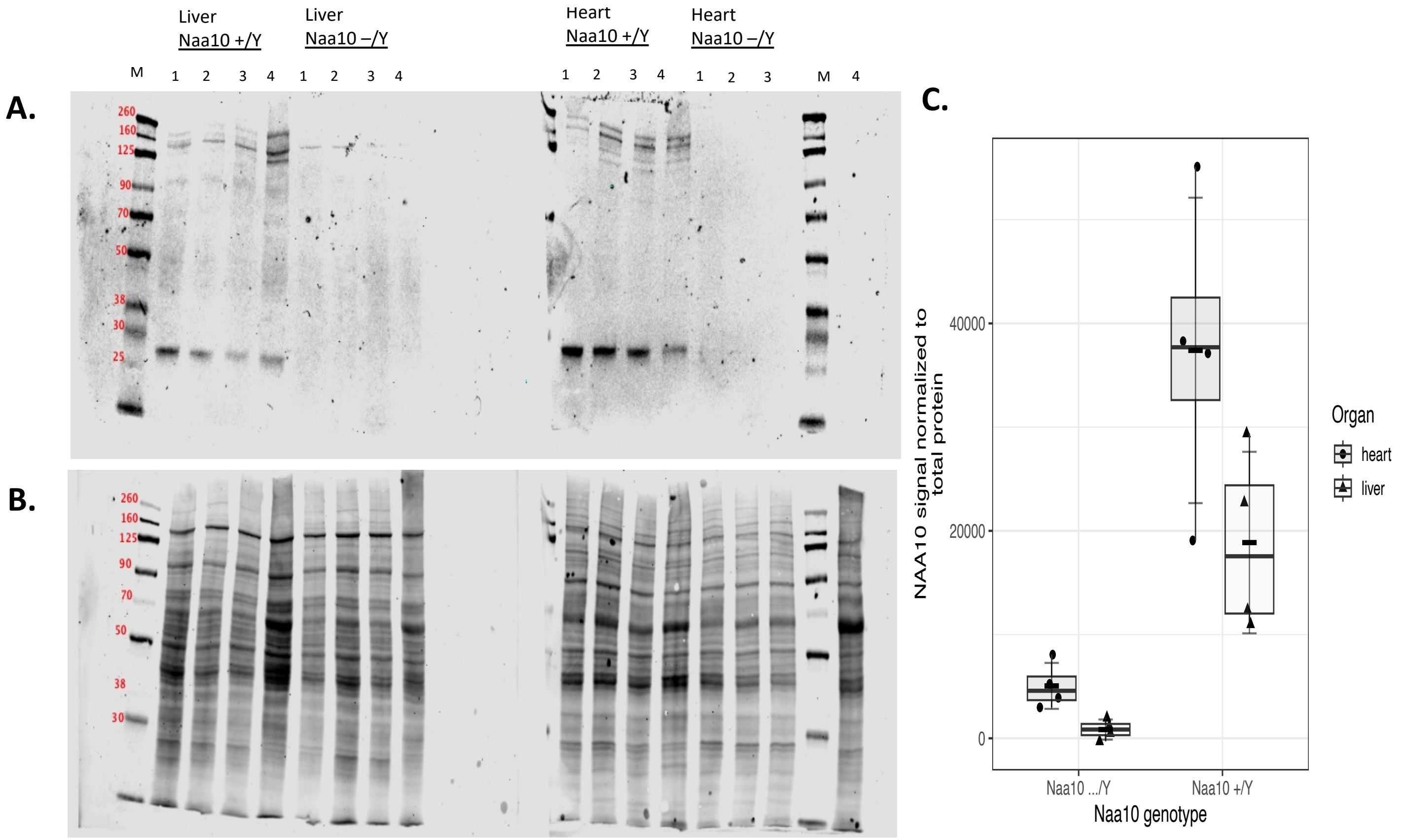

S9 Fig. Quantification of *Naa10*$^{-/Y}$ and *Naa10*$^{+/Y}$ heart and liver tissue lysate.

A) Membranes incubated in rabbit anti-NAA10 MAb and goat-anti-rabbit secondary (800 nm channel). Biological replicates (n = 8) were obtained of *Naa10*$^{-/Y}$ (N = 4) and *Naa10*$^{+/Y}$ (N = 4) mice. Heart and tissue lysate were obtained from each mouse. Blots were stained for total protein (REVERT 700 Total protein stain) post-transfer. After stain removal, blots were incubated in anti-NAA10 MAb and anti-rabbit secondary antibody. NAA10 signal was normalized to total protein. **B)** Membranes stained for total protein using REVERT 700 total protein stain (700 nm channel) to verify transfer and equal loading for NAA10 signal normalization. **C)** Quantification of normalized NAA10 signal in heart and liver lysate; horizontal crossbar indicates mean (± SD; 2-way ANOVA, F statistic = 14.52 on 3 and 12 DF, *P < 0.05)

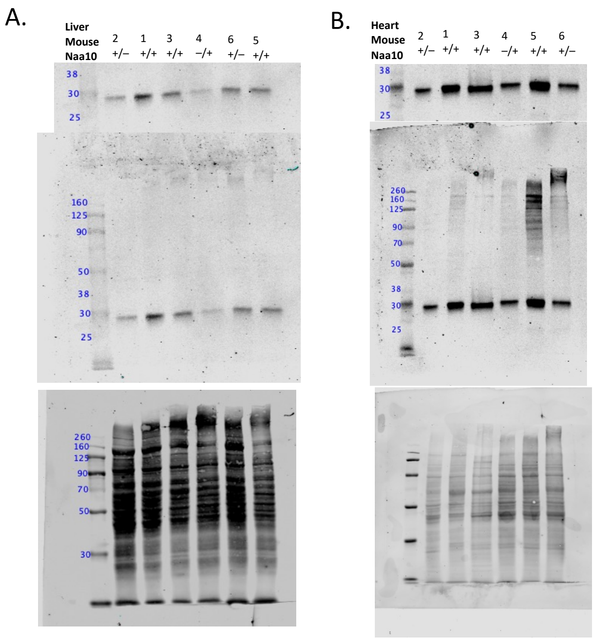

S10 Fig. NAA10 immunoblotting in heterozygous females. Whole membranes corresponding to representative immunoblots of liver and heart lysates in Figure 1. Membranes were stained for total protein after transfer using REVERT 700 total protein stain. After total protein stain removal, membranes were incubated in rabbit anti-NAA10 MAb and goat-anti-rabbit secondary antibodies. From top to bottom, target protein (NAA10) and loading control (total protein). **A)** Representative blot for immunoblotted liver lysate. From top to bottom, NAA10 excerpt, NAA10 whole membrane, and total protein stained membrane. **B)** Representative blot for immunoblotted heart lysate. From top to bottom, NAA10 excerpt, NAA10 whole membrane, and total protein stained membrane.

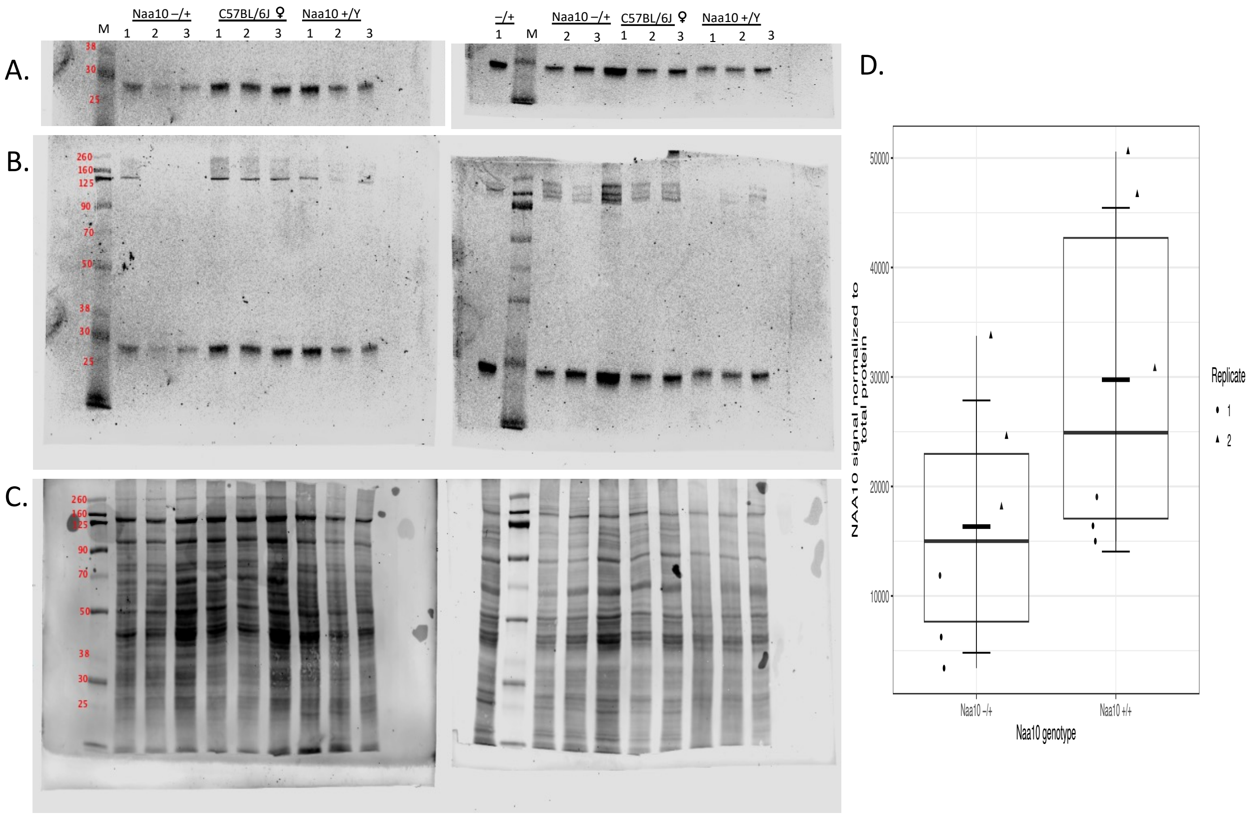

S11 Fig. Western blot analysis of NAA10 signal in heterozygous females and C57 controls. Liver lysates were obtained from *Naa10*$^{-/+}$ mice (N = 3) and C57BL/6J females (N = 3). *Naa10*$^{+/Y}$ lysates were loaded to balance gels and excluded from analysis. Both replicates are shown. Blots were stained for total protein post-transfer and scanned to verify successful transfer of equal loading for use as loading control; after total protein stain removal, blots were incubated with anti-NAA10 MAb and anti-rabbit secondary. **A-B)** NAA10 lanes and whole membranes from replicate blots incubated with anti-NAA10 Mab and goat-anti-rabbit secondary antibody. Immunostained membranes were scanned in 800nm channel. **C)** Whole membrane after post-transfer staining for total membrane using REVERT 700 total protein stain to verify transfer and equal loading for NAA10 signal normalization. Total-protein-stained membranes were scanned in 700nm channel. **D)** Quantification of NAA10 signal normalized to total protein. Short black crossbar indicates mean NAA10 signal normalized to total protein (±SD, Welch's 2-sample t-test, t = -1.6784, df = 9.1762, P = 0.1252).
