# Supplementary material for: Evaluating possible maternal effect lethality and genetic background effects in Naa10 knockout mice
Source: PLoS One. 2024 May 7;19(5):e0301328. doi: 10.1371/journal.pone.0301328 (PMC11075865; doi:10.1371/journal.pone.0301328)
Supplement: Attachment — Submitted filename: Response to reviewers 10-22-23 .docx [file pone.0301328.s003.docx]

October 21, 2023

Rebuttal letter.

PONE-D-23-22896

Evaluating possible maternal effect lethality and genetic background effects in Naa10 knockout mice

PLOS ONE

Journal requirements:

When submitting your revision, we need you to address these additional requirements.

1. Please ensure that your manuscript meets PLOS ONE's style requirements, including those for file naming. The PLOS ONE style templates can be found at

<https://journals.plos.org/plosone/s/file?id=wjVg/PLOSOne_formatting_sample_main_body.pdf> and

<https://journals.plos.org/plosone/s/file?id=ba62/PLOSOne_formatting_sample_title_authors_affiliations.pdf>

**We have gone through and updated the formatting.**

2. To comply with PLOS ONE submissions requirements, in your Methods section, please provide additional information regarding the experiments involving animals and ensure you have included details on (1) methods of sacrifice, (2) methods of anesthesia and/or analgesia, and (3) efforts to alleviate suffering.

- **Added more background information on animal welfare and our end point intervention protocols which include methods of sacrifice, anesthesia/analgesia, and effects to alleviate suffering.**

3. PLOS ONE now requires that authors provide the original uncropped and unadjusted images underlying all blot or gel results reported in a submission’s figures or Supporting Information files. This policy and the journal’s other requirements for blot/gel reporting and figure preparation are described in detail at <https://journals.plos.org/plosone/s/figures#loc-blot-and-gel-reporting-requirements> and <https://journals.plos.org/plosone/s/figures#loc-preparing-figures-from-image-files>. When you submit your revised manuscript, please ensure that your figures adhere fully to these guidelines and provide the original underlying images for all blot or gel data reported in your submission. See the following link for instructions on providing the original image data: <https://journals.plos.org/plosone/s/figures#loc-original-images-for-blots-and-gels>.

In your cover letter, please note whether your blot/gel image data are in Supporting Information or posted at a public data repository, provide the repository URL if relevant, and provide specific details as to which raw blot/gel images, if any, are not available. Email us at [plosone@plos.org](mailto:plosone@plos.org) if you have any questions.

**The uncropped images in support of the Western blotting shown in Figure 1 of the manuscript are shown now in S9, S10 and S11 in supplementary information.**

4. We note that Figures 4,6 and supplemental figure 12 in your submission contain copyrighted images.

We require you to either (1) present written permission from the copyright holder to publish these figures specifically under the CC BY 4.0 license, or (2) remove the figures from your submission:

- **Fixed figures 4, 6 and supplementary figure 12 copyright issues by fully removing ruler and adding accurate scale bars for each image using Fiji ImageJ software. In this case we will not have to obtain permission from copyright holders**

5. Please review your reference list to ensure that it is complete and correct. If you have cited papers that have been retracted, please include the rationale for doing so in the manuscript text, or remove these references and replace them with relevant current references. Any changes to the reference list should be mentioned in the rebuttal letter that accompanies your revised manuscript. If you need to cite a retracted article, indicate the article’s retracted status in the References list and also include a citation and full reference for the retraction notice.
 **We have done this. We are not aware of having cited any retracted article. We did add some new references in the Discussion (63-71), and we have re-numbered accordingly.**

Reviewers' comments:

Reviewer's Responses to Questions

**Comments to the Author**

1. Is the manuscript technically sound, and do the data support the conclusions?

The manuscript must describe a technically sound piece of scientific research with data that supports the conclusions. Experiments must have been conducted rigorously, with appropriate controls, replication, and sample sizes. The conclusions must be drawn appropriately based on the data presented.

Reviewer #1: Yes

Reviewer #2: Partly

2. Has the statistical analysis been performed appropriately and rigorously?

Reviewer #1: No

Reviewer #2: Yes

3. Have the authors made all data underlying the findings in their manuscript fully available?

The [PLOS Data policy](http://www.plosone.org/static/policies.action#sharing) requires authors to make all data underlying the findings described in their manuscript fully available without restriction, with rare exception (please refer to the Data Availability Statement in the manuscript PDF file). The data should be provided as part of the manuscript or its supporting information, or deposited to a public repository. For example, in addition to summary statistics, the data points behind means, medians and variance measures should be available. If there are restrictions on publicly sharing data—e.g., participant privacy or use of data from a third party—those must be specified.

Reviewer #1: Yes

Reviewer #2: Yes

4. Is the manuscript presented in an intelligible fashion and written in standard English?

PLOS ONE does not copyedit accepted manuscripts, so the language in submitted articles must be clear, correct, and unambiguous. Any typographical or grammatical errors should be corrected at revision, so please note any specific errors here.

Reviewer #1: Yes

Reviewer #2: Yes

5. Review Comments to the Author

Please use the space provided to explain your answers to the questions above. You may also include additional comments for the author, including concerns about dual publication, research ethics, or publication ethics. (Please upload your review as an attachment if it exceeds 20,000 characters)

Reviewer #1: The manuscript entitled „Evaluating possible maternal effect lethality and genetic background effects in Naa10 knockout mice" is a well-designed and comprehensive analysis of a novel NAA10 inbred line generated in this study. The authors focus on replicating phenotypes described in previous studies and evaluating these effects. The results are well-described and clearly ordered. The authors could confirm the majority of previously described NAA10 impacts. The previously suggested "maternal effect lethality for heterozygous Naa10-/X female mice” was unreproducible, consistent with other authors' identifying a mice-specific NAA12 variant compensating for the loss of NAA10.

Minor concerns and suggestions for manuscript improvement
- The applied statistical tests are not mentioned in all cases. A section describing the statistical analyses is missing in the material and methods.

**Added a new section to Materials and Methods to discuss all statistical tests performed within this manuscript called “Statistical Analysis”. Further elaboration on statistical testing was also added here.**

- PAGE 31 Pax3 mutant is not introduced. Please explain for the non-expert reader
- Page 31 “As such, there is modulation of these neural crest-related phenotypes by genetic background. There is a….” Rephrase these statements and the most relevant primary literature

**We have added this as follows:**

**" We have previously noted that *Naa10^-/-^* mutants somewhat phenocopy *Pax3* mutants [21]. *Pax3* knockout mice, which model symptoms of Waardenburg syndrome, lack a transcription factor necessary for cardiac neural crest cell (cNCC) migration and proliferation in the pharyngeal arches and embryonic outflow tract. Without Pax3, inadequate NCC migration results in congenital heart defects attributable to improper remodeling of the arches and outflow tract [72–75]. *Pax3^+/-^* adults have 100% piebaldism, along with variable penetrance for neural crest (NC)-related persistent truncus arteriosus (PTA) or double outlet right ventricle (DORV) with concomitant ventricular septal defects VSDs [72,73,76,77], congenital hydrocephalus [78], and/or skeletal defects due to abnormal somite morphogenesis [79,80]."**

- Discussion: The discussion could start with a broader perspective on NAA10 function in animals and other organisms (plants, fungi) and summarize the existence of NAA10 homologs in these species. This would help the non-expert readers to classify the here described findings in the context of NatA evolution.

**We have added a new paragraph at the beginning of the Discussion, as follows:**

***NAA10*, which encodes the catalytic subunit of the principal NAT complex (NatA) and is mutated in humans affected by OS, is likely an essential gene for eukaryotic development. Initially reported in yeast, other Naa10 (also known as Ard1) orthologues were later identified in humans and various model organisms, including *D. melanogaster, D. rerio, A. thaliana*, and *M. musculus* [55,56]. In *D. melanogaster*, ovary-specific loss of Naa10 causes defective oogenesis in *vnc* mutants. The loss of Naa10 also contributes to cell growth and proliferation failure in wing discs [57]. Morpholino-mediated Naa10 knockdown in *D. rerio* increases developmental lethality; further, surviving zebrafish exhibit growth failure and other developmental abnormalities (e.g., bent axes, missing eyes, and missing tails) [58]. In addition to the apparent conservation of Naa10, the NTA profile of plants and animals are broadly similar which supports the necessity of this protein modification in eukaryotes [59,60]. *Naa10* knockout mouse lines also support the necessity of NatA-catalyzed NTA in mammalian development [21,61,62]. Previously, we showed that Naa12 complexes with Naa15 to form functional NatA in Naa10^–/Y^ animals, which explains why these mice were born and were not completely embryonic lethal, at least in the mixed genetic background reported in that paper [21]. Confirming this, double mutants for Naa10 and Naa12 knockout mice were embyronic lethal [21].**

Reviewer #2: In this manuscript, the authors tried to develop a mouse model for Ogden Syndrome with a Ser37Pro missense mutation by using two approaches. However, the gene editing did not work as they expected, and they obtained new knockout mice for Naa10. The authors found the new Naa10 knockout mice show similar phenotypes as previously reported (Kweon et al. 2021). Moreover, they demonstrated these phenotypes are modulated by genetic background and environmental effects. The reviewer has the following concerns for this manuscript.

Major concerns

1. In this manuscript, the authors observed embryonic lethality for some Naa10 knockout mice that were not reported in the previous publication (Kweon et al. 2021). The authors reasoned it is probably due to genetic backgrounds and/or environmental effects. The authors also stated it in the abstract as a new discovery for this manuscript. However, there are no data supporting this statement. The authors should perform some experiments such as examination of NatA enzyme activity to confirm NatA activity is influenced by the genetic backgrounds and environmental conditions in the new knockout mice.

**The reviewer refers to "new knockout mice", but it is not clear if they mean the prior allele from our prior ELife paper that has now been back-crossed 20 generations, or if they are referring to the minigene or indel mice reported in the current paper too. However, the minigene and indel mouse lines are no longer alive, as they were euthanized, and we do not have the funding needed to resurrect these lines from the cryogenically preserved sperm for these strains.**

**For the prior allele (with the neomycin cassette, as reported in our prior ELife paper), after 20 back-crosses, there is now a small amount of embryonic lethality in males of the previously published mouse strain on a pure genetic background and in a new mouse facility, as presented in the Results section of the manuscript. In the Discussion section, we present several hypothetical mechanisms associated with gene targeting methodology and potential maternal stress effects to the change of colony venue (independent of NatA function), along with supporting literature, that could potentially underlie this phenomenon. However, it is beyond the scope of our currently funded project and paper to attempt to find the exact explanation for why about 25% of the male mice are now dying during embryogenesis on the pure genetic background and in the new IBR facility. We do have unpublished data with Jackson laboratory in which we have generated a frameshift in Naa10 by introducing a new 2-base pair indel in Naa10, using CRISPR technology, with those mice having been made at Jackson Laboratory in Maine in genetically inbred C57BL/6J zygotes. We plan to publish these new mice in a separate paper, but the results thus far demonstrate that those mice do not have any embryonic lethality in the mouse facility at JAX. As such, knocking out Naa10 by just a 2-base pair indel on an inbred C57BL/6J genetic background does not cause any embryonic lethality. Therefore, it seems that it is more likely that the 25% embryonic lethality that we are observing at IBR must be related to either something with the neomycin cassette from the gene targeting (which itself might be modulated by genetic background or environment), or otherwise something with maternal stress in the IBR mouse facility. For now, we are reporting the results at hand, where we have genotyped all embryos at least twice to ensure accuracy, and the Discussion proposes the possible explanations, including genetic background, as we are not prepared yet to publish the new JAX mice, as there is substantial work remaining to fully characterize that new line.**

**We do not plan to assess NatA enzyme activity directly in the mouse embryos of the currently available line back-crossed >20 generations, as it would be quite difficult to do this in embryos, where only 25% of the males are dying and in which we cannot predict which of those embryos will actually die, as there is no gross anatomical measurement that reliably predicts which ones will die and which ones will not. Even if we somehow could predict which ones will die, we then calculate that we would have to dissect ~ 100 embryos, genotype them, make embryonic lysates from them, and then assess enzymatic activity in those 100 lysates on some substrate peptide, in order to prove that the 25% of Naa10 knockout males predicted to die (n~6) somehow have statistically significantly less NatA activity than the 75% of males that are predicted to not die (n~18), compared to male and female littermate controls (n~75). This has never been done before, to our knowledge, with mouse embryo lysates, and setting up such an assay is simply beyond the scope of the currently funded project, as it could easily take us more than one year to dissect out 100 embryos, discover a way to reliably predict which ones will die, and then develop an assay that is sensitive enough to enable us to detect significant differences in NatA activity. Please note that Figure 3 of our prior paper in ELife included immunoprecipitation of the NatA complex from mouse tissues (n=3 for male WT and n=3 for male KO), followed by enzymatic activity assay on a substrate peptide, just to prove that there was enzymatic activity present, postulated to originate from Naa12. However, to do this with statistical rigor on 100 mouse embryo lysates is quite complex, and there is no way of knowing if the immunoprecipitation step might be affected by the genetic background, environment, or neomycin cassette, either. As such, direct testing of the enzymatic lysates without immunoprecipitation would eliminate that variable, but as stated, this has never been done successfully before. As such, what the reviewer is asking for is outside the scope of what is possible for this current paper.**

**For the abstract, we are not claiming to know the mechanism of this small embryonic lethality, and the abstract currently reads as follows:**

**"We cannot replicate a prior report of "maternal effect lethality" for heterozygous *Naa10^-/X^* female mice, but we do observe a small amount of embryonic lethality in the *Naa10^-/y^* male mice on the inbred genetic background in this different animal facility."**

**We have also added the following to the Discussion, in an attempt to better address this concern of the reviewer:**

**" The present study examined this on an inbred C57BL/6J genetic background (20 backcrosses) and in the new environment of the animal facility at IBR, where we did observe some embryonic lethality (~20-25% of *Naa10^-/Y^* male mice), even in the presence of *Naa12*. Phenotypic variation due to genetic background has been observed in several genetically engineered mouse models [63]. Alternatively, the transfer of all mice to a new animal colony and environment in March 2019 may have contributed to this embryonic lethality potentially through the effects of maternal stress on *in utero* development [64] or immune system dysfunction [65]. In support of this latter hypothesis, there was a parallel increase in neonatal lethality in the new colony observed for all mice, including the wild type *C57BL/6J* mice (unpublished data). Lastly, the gene targeting scheme presents the possibility that insertion of the neomycin cassette utilized to generate this strain of Naa10 KO mice may have resulted in dysregulation of off-target gene expression - a phenomenon documented in previously published literature [66–71], which could itself be modified by genetic background and environment, although this is not something that we have investigated for our mice."**

2. The cardiac diseases are a common developmental anomaly in Ogden Syndrome patients. The abnormal cardiac development was reported in the previous Naa10 knockout mice (Kweon et al. 2021). The authors should examine the cardiac development in the new Naa10 knockout mice.

**It is not clear if the reviewer is referring to the minigene mice, indel mice, or both strains? However, in either case, the minigene and indel mouse lines are no longer alive, as they were euthanized, and we do not have the funding needed to resurrect these lines from the cryogenically preserved sperm for these strains. Furthermore, as we reported in the manuscript, those lines were on a mixed genetic background, and we saw no embryonic or neonatal lethality. We did perform necropsy on these lines for Western blotting, including dissecting out heart tissues. There was no obvious defect observed with these hearts on gross anatomy inspection, and this is expected given the fact that there was not any embryonic or neonatal lethality. It is possible that there might have been sub-clinical manifestations, such as a ventricular (VSD) or atrial septal defect (ASD), but such a thing could only be detected by either echocardiography or with serial histology sectioning of hearts. Please note that in Figure 1D of the previously published ELife paper, the quantitation of VSD and ASD in E18.5 embryos was only 6/20 and 7/20, respectively, and therefore the presence of this phenotype in the minigene and indel mice is likely much less than this. As such, we did not think it was worth spending the time and money to do this in the minigene or indel mice, as there was clearly no embryonic or neonatal lethality to explain. Although we appreciate that some of the humans with Ogden syndrome have been found to have VSD or ASD, we prefer to focus our future efforts on the female mice that are on a genetically inbred background and which are either compound heterozygous for Naa10 and Naa12 or heterozygous for Naa10 and homozygous null for Naa12 (if any of those mice survive). Such mice are a much better model for the human disease, and we expect they will have much higher penetrance for many of the symptoms found in humans, including cardiac issues. Such experiments are ongoing and will be reported in a future paper. We hope that this information helps to explain to the reviewer why we did not examine cardiac development in the minigene or indel mice.**

Minor concerns

In the Materials and Methods, the authors used GraphPad Prism 6 to plot the data for measurement of piebaldism. But in the Data Analysis, the authors stated Prism 9 Graphpad software version 9.5.1 was used. Why did the authors use two versions of the software?

**Fixed this to version 9.5.1 for all analyses in prism since all statistical test were reran using version 9.5.1.**

6. PLOS authors have the option to publish the peer review history of their article ([what does this mean?](https://journals.plos.org/plosone/s/editorial-and-peer-review-process#loc-peer-review-history)). If published, this will include your full peer review and any attached files.


If you choose “no”, your identity will remain anonymous but your review may still be made public.


**Do you want your identity to be public for this peer review?** For information about this choice, including consent withdrawal, please see our [Privacy Policy](https://www.plos.org/privacy-policy).

Reviewer #1: No

Reviewer #2: No

[NOTE: If reviewer comments were submitted as an attachment file, they will be attached to this email and accessible via the submission site. Please log into your account, locate the manuscript record, and check for the action link "View Attachments". If this link does not appear, there are no attachment files.]

While revising your submission, please upload your figure files to the Preflight Analysis and Conversion Engine (PACE) digital diagnostic tool, <https://pacev2.apexcovantage.com/>. PACE helps ensure that figures meet PLOS requirements. To use PACE, you must first register as a user. Registration is free. Then, login and navigate to the UPLOAD tab, where you will find detailed instructions on how to use the tool. If you encounter any issues or have any questions when using PACE, please email PLOS at [figures@plos.org](mailto:figures@plos.org). Please note that Supporting Information files do not need this step.

**We uploaded figures to this tool, and we now have TIFF files that we can supply.**
